# ShDcR3 sensitizes TRAIL-resistant HCC cells by inducing caspase-dependent apoptosis while suppressing NF-κB dependent cFLIP_L_ expression

**DOI:** 10.1371/journal.pone.0191545

**Published:** 2018-02-14

**Authors:** Dong-Yu Liang, Wei Huang, Qing Chang, Yan-Qiang Hou

**Affiliations:** 1 Department of Central Laboratory, Songjiang Hospital Affiliated First People’s Hospital, Shanghai Jiao Tong University, Shanghai, China; 2 Department of Central Laboratory, Jiading District Central Hospital Affiliated Shanghai University of Medicine&Health Sciences, Shanghai, China; 3 Department of Blood Bank, Jiading District Central Hospital Affiliated Shanghai University of Medicine&Health Sciences, Shanghai, China; Institute of Biochemistry and Biotechnology, TAIWAN

## Abstract

Evidence has shown that most hepatocellular carcinoma (HCC) cells are resistant to tumor necrosis factor (TNF)-related apoptosis-inducing ligand (TRAIL)-mediated apoptosis. However, the molecular mechanisms underlying TRAIL-mediated apoptosis resistance are not well understood. In this study, we reported that downregulation of Decoy receptor 3 (DcR3) expression by lentiviral vectors carrying shRNA against DcR3 (LV-ShDcR3, shDcR3) in Huh7 both greatly enhanced TRAIL-mediated apoptosis and reduced cell proliferation capability. In addition, silencing DcR3 resulted in upregulation of the cell apoptotic regulators including Bid, caspase-3, and caspase-8. Caspase inhibitors inhibited shDcR3-mediated cell death, which indicated that downregulation of DcR3 expression in Huh7 cells increased TRAIL-induced caspase-dependent apoptotic cell death. Furthermore, although the knockdown of DcR3 altered the expression of some Bcl-2- and IAP-family proteins, this change was inhibited by pretreatment with a pancaspase inhibitor, which indicated the cytotoxic effect of shDcR3 was not due to the expression of these proteins. In contrast, shDcR3 significantly inhibited TRAIL-induced transcription factor nuclear κB (NF-κB) activation through the IκB kinase (IKK) pathway, as well as inhibited TRAIL-induced increases in FLICE-inhibitory protein long form (cFLIP_L_) expression at the transcriptional level. Silencing cFLIP_L_ expression mimicked the cytotoxic effect of shDcR3 on TRAIL-mediated cell apoptosis. Moreover, overexpression of cFLIP_L_ effectively prevented the increase in cell apoptosis in Huh7 cells co-treated with TRAIL and shDcR3. Taken together, our findings indicated that silencing DcR3 sensitizes TRAIL-mediated apoptosis in HCC cells by inhibiting NF-κB.

## Introduction

Hepatocellular carcinoma (HCC) accounts for 90% of all primary liver cancers: although common worldwide, it is particularly prevalent in Asia [1]. Due to its low surgical resection but high recurrence, HCC is the second leading cause of death globally [2, 3]. The balance between pro-apoptotic and anti-apoptotic factors is important in hepatocarcinogenesis. Tumor cells, through overexpression of anti-apoptotic factors in intra- and intercellular sites, tip the balance towards their own survival. Overexpression of these factors leads to the resistance of HCC cells to apoptosis, resulting in a loss of tumor growth control [4–6]. Therefore, understanding the mechanisms that restore the sensitivity of HCC cells to apoptosis could be useful for the treatment of HCC.

The death receptor pathway is an extracellular apoptosis pathway: by binding to extracellular death receptors, the extracellular pro-apoptotic ligands activate apoptotic signaling and induce apoptosis [7]. The extracellular ligands belong to the tumor necrosis factor (TNF) superfamily, and TNF-related apoptosis inducing ligand (TRAIL) is a member of the TNF superfamily, which has been demonstrated to induce apoptosis in various types of tumor cells without toxicity to normal cells [8]. However, several tumor cell lines, including HCC cell lines, exhibit resistance to TRAIL-mediated apoptosis [9–11]. TRAIL has been shown to activate not only the apoptotic signal pathway but also NF-κB, leading to the transcription of genes known to antagonize the death signaling pathway [12]. Therefore, understanding the underlying mechanisms involved in the resistance to TRAIL-induced apoptosis and restoring sensitivity to TRAIL in HCC cells could be used in the treatment of HCC.

As previously reported, decoy receptor 3 (DcR3), a soluble decoy receptor also known as TR6 or M68, is a member of the TNFR superfamily. As it lacks a transmembrane domain, DcR3 can be secreted into the extracellular space. DcR3 is located on chromosome position 20q13, which is associated with gene amplification in various types of cancer [13]. Evidence strongly indicates that DcR3 is overexpressed in a variety of tumor cells, including in adenocarcinomas of the esophagus, stomach, colon, rectum, and pancreas, in lymphomas, and in gliomas [14]. It has been shown that DcR3 competes with the binding of related ligands such as FasL, TL1A, LIGHT, and thus blocks apoptosis, impedes the immune response, and induces angiogenesis [15]. Accumulating evidence has demonstrated that members of the TNF superfamily can induce “reverse signals” after binding with their receptors [16]. DcR3 was shown to trigger a reverse signaling pathway involving phosphoinositide-3-kinase, protein kinase C, and NF-κB, to modulate other physiological or pathological effects [17]. As in HCC cells, the mechanism of resistance to TRAIL-induced apoptosis is primarily the activation of the NF-κB pathway through both the upregulation of apoptotic inhibitors such as cFLIP_L_ and the upregulation of anti-apoptotic molecules [18–20]. However, whether DcR3 affects the apoptosis of HCC cells remains to be determined. Thus, it is critical to examine the effects of DcR3 on the occurrence and progression of HCC, particularly with respect to cell apoptosis.

Here, we examined the effect of DcR3 deficiency on TRAIL-induced Huh7 cell apoptosis, and investigated its underlying mechanisms. We showed that downregulation of DcR3 in TRAIL-treated Huh7 cells increased caspase-dependent apoptosis and inhibited TRAIL-mediated NF-κB activation and cFLIP_L_ protein expression.

## Materials and methods

### Reagents and cells

Human hepatocellular carcinoma cell line Huh7 was purchased from the American Type Culture Collection (Manassas, VA, USA); cultured in Dulbecco’s Modified Eagle’s Medium (DMEM) supplemented with 10% fetal bovine serum (FBS), 100 U/mL penicillin, and 100 μg/mL streptomycin; and maintained in a humidified incubator with 5% CO_2_ at 37°C. Human hepatocellular carcinoma cell line HepG2 was purchased from the American Type Culture Collection (Manassas, VA, USA); cultured in Roswell Park Memorial Institute -1640 (RPMI-1640) supplemented with 10% FBS, 100 U/mL penicillin, and 100 μg/mL streptomycin; and maintained in a humidified incubator with 5% CO_2_ at 37°C. RPMI-1640, DMEM, FBS, penicillin, and streptomycin were obtained from Invitrogen (Carlsbad, CA, USA). Recombinant TRAIL was purchased from R&D Systems (Minneapolis, MN, USA) and dissolved in DMSO. For each experiment, in the group, which was not treated with TRAIL, DMSO was used as control. All antibodies were purchased from Cell Signaling Technology, Inc. (Beverly, MA, USA), except for the anti-glyceraldehyde 3-phosphate dehydrogenase (GAPDH) polyclonal antibody, which was purchased from Santa Cruz Biotechnology, Inc. (Santa Cruz, CA, USA). DcR3 antibody (#4758, 1:1000), Caspase 8 (1C12) mouse antibody (#9746, 1:1000), Caspase 3 (8G10) rabbit mAb (#9665, 1:1000), tBid (3C5) mouse mAb (#8762, 1:1000), Cytochrome C (D18C7) rabbit mAb (#11940, 1:1000), Cleaved PARP (Asp214) (D64E10) rabbit mAb (#5625, 1:1000), Bcl-xl (54H6) rabbit mAb (#2764, 1:1000), Bcl-2 (124) mouse mAb (#15071, 1:1000), Bad antibody (#9292, 1:1000), xIAP (3B6) rabbit mAb (#2045, 1:1000), cIAP1 antibody (#4952, 1:1000), cIAP2 (58C7) rabbit antibody (#3130, 1:1000), cFLIP_L_ (D5J1E) rabbit mAb (#56343, 1:1000), p65 (D14E12) rabbit mAb (#8242, 1:1000), P-IKK (Ser176/180) rabbit mAb (#2697, 1:1000), p-IKB (14D4) rabbit mAb (#2859, 1:1000) and GAPDH antibody (sc47724, 1:1000). IETD, DEVD, and z-VAD were purchased from Alexis (SanDiego, CA, USA). Caspase-8 and caspase-3 activity assay kits were purchased from Nanjing Jiancheng Biological Co., Ltd. (Nanjing, China).

### Quantitative Real time PCR (qPCR)

We used TRIzol^®^ reagent (Invitrogen, Carlsbad, USA) to isolate total RNA from the target cells and tissues, and generated cDNA with a ReverTra Ace^®^ qPCR RT kit (FSQ-101; Toyobo, Osaka, Japan). A SYBR^®^ Premix Ex Taq™ (Takara, Chiga, Japan) kit and a LightCycler (7500; Applied Biosystems^™^, Foster City, CA, USA) were used for qPCR analyses. Reactions were performed under the following conditions: 95°C for 30 sec, followed by 40 cycles of 95°C for 5 sec and 60°C for 30 sec. We confirmed the amplification specificity of each reaction with a melting curve. GAPDH was used as a control housekeeping gene. The relative expression levels were normalized to GAPDH and calculated using the equation 2^–ΔΔCt^ (ΔCt = Ct-target– Ct-GAPDH, ΔΔCt = ΔCt experiment group-ΔCt control group). All experiments were performed at least three times.

### Cell culture and infection

Huh7 or HepG2 cells were seeded at a density of 5 × 10^4^ cells/well in six-well plates. The following day, in the presence of 5 μg/mL polybrene, cells were infected with either 10 μL recombinant lentivirus containing DcR3 shRNA (ShDcR3; experimental group) or 10 μL mock lentivirus (LV-NC; control group). The lentiviral vectors carrying shRNA against DcR3 were constructed by the Heyuan Biotechnology Company (Shanghai, China). The sequence for shRNA DcR3 was 5’-TCATCGACTTTGTGGCTTT-3’. Each experiment the group which was not treated with shDcR3, the LV-NC was used as control.

### Western blotting

Cells were washed in phosphate-buffered saline (PBS) three times and incubated on ice in lysis buffer for 30 min. For cytochrome c detection, the collected cells were first homogenized in ice-cold mitochondrial fractionation buffer (Biyuntian, Shanghai, China). Then the homogenates were centrifuged for 20 min at 10000 g. The supernatants were the cytosolic fractions. After being boiled in sodium dodecyl sulfate (SDS) loading buffer (Biyuntian, Shanghai, China), equal amounts of protein were subjected to SDS-PAGE (Biyuntian, Shanghai, China) and transferred to nitrocellulose membranes (Millipore, Tullagreen, Ireland). Each membrane was then blocked in 5% nonfat dried milk containing 0.1% Tween 20 for 1 h at room temperature, and incubated at 4°C overnight with the specific primary antibodies. The membrane was then washed in PBS three times and further incubated with horseradish peroxidase (HRP)-conjugated secondary antibody for 2 h at room temperature. After a final wash, we visualized immunoreactive bands with ECL reagent (Thermo-Fisher Scientific, Rockford, IL, USA).

### Cell viability assay

Cell viability was measured with the Cell Counting Kit-8 (CCK-8) (Dojindo Laboratories, Kumamoto, Japan) as follows. Cells were seeded in 96-well plates at a density of 5 × 10^3^ cells/well and cultured in 5% CO_2_ at 37°C until very dense colonies grew. Each group of cells was then incubated a further 48 h with various treatments. Finally, 20 μL CCK-8 (Dojindo Laboratories, Kumamoto, Japan) was added to each well and incubated at 37°C for an additional 2 h. Absorbance of each well was detected at a wavelength of 450 nm by an ultraviolet spectrophotometer (Thermo-Fisher Scientific, Rockford, IL, USA) and cell viability was assessed as follows: (experimental OD/control OD) x 100%.

### Analysis of apoptosis with flow cytometry

Huh7 cells were organized into four groups on a six-well plate; each group was incubated with a different treatment. After 24 h, the cells were washed twice using ice cold PBS, and harvested with binding buffer. After the addition of 0.5 mg/mL fluorescein isothiocyanate (FITC)-Annexin V and 0.6 mg/mL Propidium iodide (PI) of the cell suspension, the mixture was incubated in the dark at room temperature for 15 min. Stained cells were immediately analyzed with FASCalibur and CellQuest (Becton Dickinson, San Jose, CA, USA). Cells positive for annexin V were considered apoptotic cells. All samples were assayed in triplicate.

We measured the activity of caspase-8 and caspase-3 with a caspase-8 and caspase-3 activity assay kit (Jiancheng, Nanjing, China) following the manufacturer's instructions. Briefly, cells were lysed using lysis buffer for 15 min at 4°C, then centrifuged at 16,000 x g for 15 min. The supernatants were incubated with Ac-IETD-pNA or Ac-DEVD-pNA for 60–120 min at 37°C. Caspase activity was determined by measuring the proteolytic cleavage of the chromogenic substrates Ac-IETD-pNA or Ac-DEVD-pNA at 405 nm.

### Nuclear and cytoplasmic proteins extraction

Nuclear and cytoplasmic proteins were obtained using a NE-PER Nuclear and Cytoplasmic Extraction kit (Thermo-Fisher Scientific, Rockford, IL, USA). Briefly, after infection with shDcR3 for 12 h in the presence of 100 μ M z-VAD-fmk, Huh7 or HepG2 cells were treated with TRAIL (75ng/ml) for 24 h. The cells were then digested and centrifuged for 10 min. The resulting pellet was collected and suspended in Cytoplasmic Extraction Reagent I (CERI) for 10 min on ice followed by the addition of ice-cold CERII. After vortexing and 10 min of centrifugation, the supernatant containing the cytoplasmic extract was collected in a clean tube and stored at -80°C. The pellet containing the nuclei was resuspended in ice-cold Nuclear Extraction Reagent (NER). After vortexing and centrifugation, the supernatant containing the nuclear extract was collected into another clean tube and stored at -80°C.

### Confocal microscopy

After infection with shDcR3 for 12 h in the presence of 100 μ M z-VAD-fmk, Huh7 cells were treated with TRAIL (75ng/ml) for 24 h. Then the cells were washed in PBS once, fixed with 4% formaldehyde for 30 min at room temperature, washed in PBC again, and permeabilized with 0.1% Triton X-100 for 5 min. After blocking with blocking buffer (1% bovine serum albumin) for 1 h, the cells were incubated with anti-p65 antibody (1:400 dilution) overnight at 4°C. The cells were incubated in Alexa Fluor 488-conjugatedanti-rabbit secondary antibody (1:200) for 2 h, followed by DAPI for 5 min. After the incubation, the cells were washed with PBS three times. Finally, the cells were observed under a confocal laser scanning microscope (Leica, TCSSP2; Leica, Wetzlar, Germany).About 10 cells were randomly selected for each experiment, and three independent experiment were performed. The fluorescence intensities were detected using a single cell level.

### Luciferase reporter assays

After infection with shDcR3 for 12 h in the presence of 100 μM z-VAD-fmk, Huh7 or HepG2 cells were treated with TRAIL (75ng/ml) for 24 h. Then, a mixture of 1 μg of NF-κB promoter-luciferase construct and 3ul Lipofectamine 2000 resuspended in 100ul Dulbecco's modification of Eagle's medium (DMEM) was added to the cells. The cells were lysed with reporter lysis buffer 24 h after transfection and luciferase activity was detected using the Luciferase Assay System (Promega, USA) following the manufacturer’s protocol. The NF-κB promoter-luciferase plasmid was purchased from Heyuan Biotechnology (Shanghai, China). It contains two NF-κB responsive elements.

### cFLIP_L_ silence and overexpression

For the cFLIP_L_ silence assay, Huh7 cells were seeded at a density of 4 × 10^4^ cells/well in six-well plates. After 24 h, 50 nM siRNA was transfected into the cells using Lipofectamine 2000. Six hours after initial incubation, the medium was removed, fresh medium was added, and the plates were incubated for further study. The sequence for cFLIP_L_ siRNA was 5′-AAGATGAAGAGCAAGCCCCTA-3′. For the overexpression assay, Huh7 cells were seeded at a density of 5 × 10^4^ cells/well in six-well plates. After 24 h, cells were transfected with either the control vector (pcDNA3) or the vector containing cFLIP_L_ using lipofectamine 2000. After an additional 24 h, the cells were either lysed or incubated for further experimentation.

### Statistical analysis

All data are expressed as mean ± SEM. Intergroup comparisons were evaluated using the one-way analysis of variance. The data were analyzed with SPSS, version 16.0 (SPSS, Chicago, IL, USA). We considered P < 0.05 to be statistically significant.

## Results

### Downregulation of DcR3 sensitizes Huh7 cells to TRAIL-induced apoptosis

**Fig 1:**
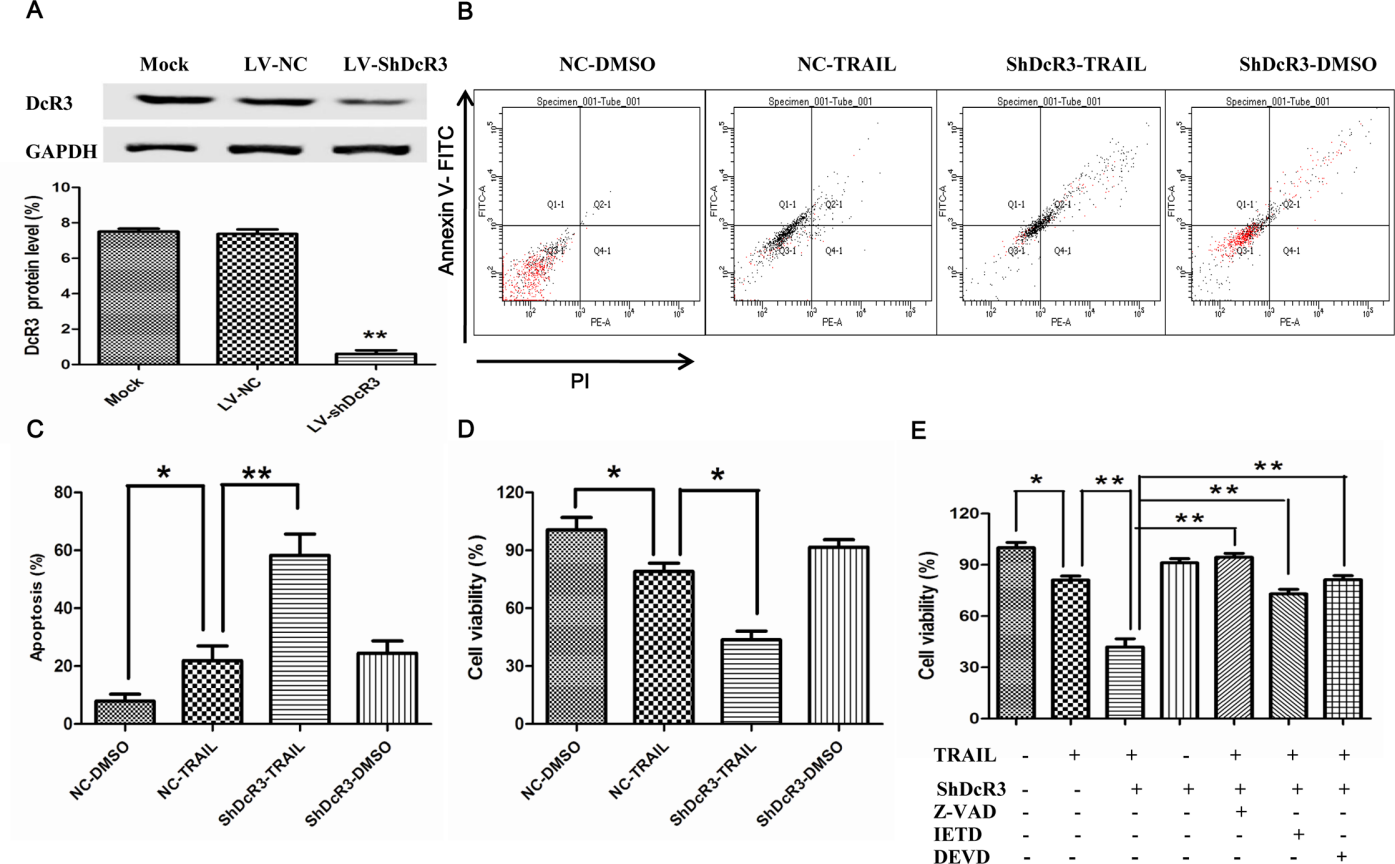
Downregulation of DcR3 increases TRAIL-induced apoptotic cell death. After infection with shDcR3 for 24 h, Huh7 cells were treated with TRAIL (75ng/ml) for 24 h. (A) DcR3 protein was detected with western blots in Huh7 cells. (B) Cell apoptosis was detected by flow cytometry in Huh7 cells. (C) The percent of apoptosis cell detected by Huh7 cells. (D) After silencing DcR3 expression, cell viability was examined by CCK-8 assays at 48 h post shDcR3 infection in Huh7 cells. (E) The effect of downregulation of DcR3 on TRAIL-induced cell viability was examined by CCK-8 assay in the presence or absence of 100 μ M of caspase inhibitors. Each value represents the mean ± SEM of three independent experiments performed in triplicate. *P<0.05.

To explore the function of DcR3 in cell apoptosis mediated by TRAIL in Huh7 cells, we first designed and constructed shDcR3 to suppress the expression of DcR3 in Huh7 cells. Our results suggest that the expression of DcR3 is reduced following the shDcR3 infection of Huh7 cells (Fig 1A).

It is well known that the main function of TRAIL is to induce apoptosis, and most HCC cells are resistant to TRAIL-induced apoptosis. Here, we examined whether downregulation of DcR3 increased TRAIL-induced apoptosis in Huh7 cells. Flow cytometry showed that treatment with TRAIL alone resulted in a few Huh7 cells death, while in cells combined treatment with TRAIL and shDcR3 the rate of cell death increased to about 60% (Fig 1B and 1C). Cell proliferation, as determined by CCK-8 assay, supported these results (Fig 1D).

To further investigate whether cell death induced by shDcR3 through caspases, we used caspase inhibitors. We found that cell death was further strongly inhibited by caspase inhibitors (Fig 1E). These results indicate that the downregulation of DcR3 expression sensitizes Huh7 cells to TRAIL-induced apoptosis through a caspase-dependent mechanism.

### Downregulation of DcR3 activates TRAIL-mediated caspase cascade

**Fig 2:**
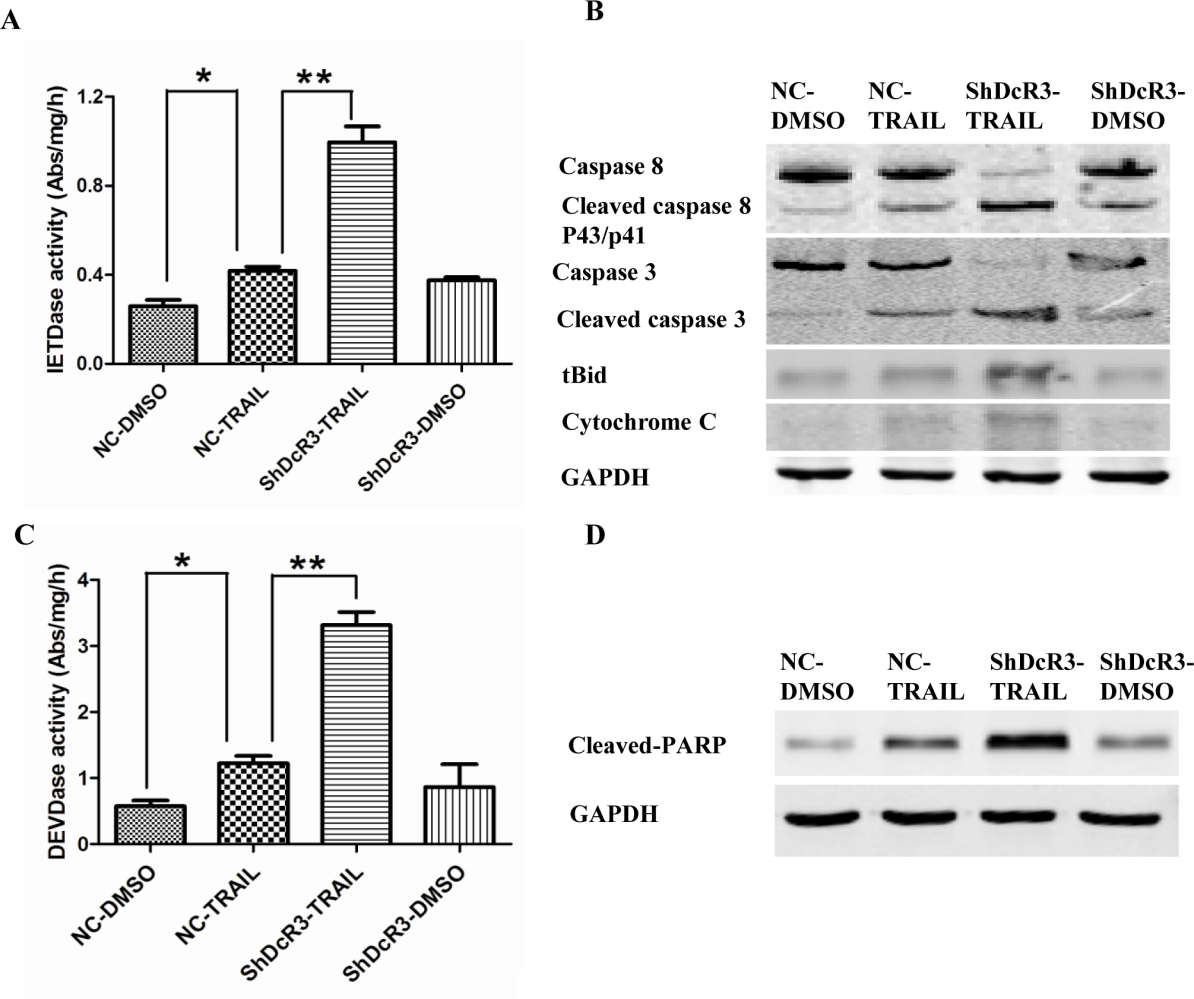
Effects of DcR3 knockdown on TRAIL-induced apoptotic signal activation. After infection with shDcR3 for 24 h, Huh7 cells were treated with TRAIL (75ng/ml) for 24 h. (A) IETDase (caspase-8-like enzyme) activities in the cell lysates were detected by colorimetric assays. (B) Caspase-8 activation, Bid cleavage, cytochrome c release, and caspase-3 activation in the cell lysates were detected by western blot. (C) DEVDase (caspase-3-like enzyme) activities in the cell lysates were detected by colorimetric assays. (D) The marker protein of apoptosis, PARP, was detected by western blot in the cell lysates. Each value represents the mean ± SEM of three independent experiments performed in triplicate.*P<0.05 and **P<0.01.

To investigate whether shDcR3 induces TRAIL-mediated apoptosis via the sequential death signal cascade, we first detected the activated caspase-8-like protease (IETDase) activity. Our results suggest that TRAIL alone slightly elevated IETDase catalytic activity in experimental cells as compared to untreated control cells, and IETDase catalytic activity was further elevated in the experimental cells when co-treated with TRAIL and shDcR3 (Fig 2A and 2B). Activated caspase-8 activates the Bid protein and induces a mitochondrial membrane change that increases the release of cytochrome c. Cytochrome c, a proapoptosis signal molecule, triggers caspase-3 activation and eventually induces apoptosis. Therefore, we next investigated whether the downregulation of DcR3 would affect the cleavage of Bid, the release of cytochrome c, and the activation of the release of caspase-3 cytochrome c. Huh7 cells treated with TRAIL alone slightly increased the cleavage of Bid, and the cleavage of Bid increased further in cells co-treated with TRAIL and shDcR3. Furthermore, co-treatment with TRAIL and shDcR3 significantly increased cytochrome c release and caspase-3 activation (p<0.01; Fig 2B and 2C). Poly ADP-ribose polymerase (PARP) is an endogenous substrate of caspase-3. Treatment with TRAIL or shDcR3 alone slightly increased the cleavage of PARP, However, combined treatment significantly increased the cleavage (Fig 2D). Our results support the hypothesis that downregulation of DcR3 modulates TRAIL-induced extrinsic apoptotic signal cascades, and thereby increases TRAIL-induced cell apoptosis.

### ShDcR3 down-regulates cFLIP_L_ expression at the transcriptional level

**Fig 3:**
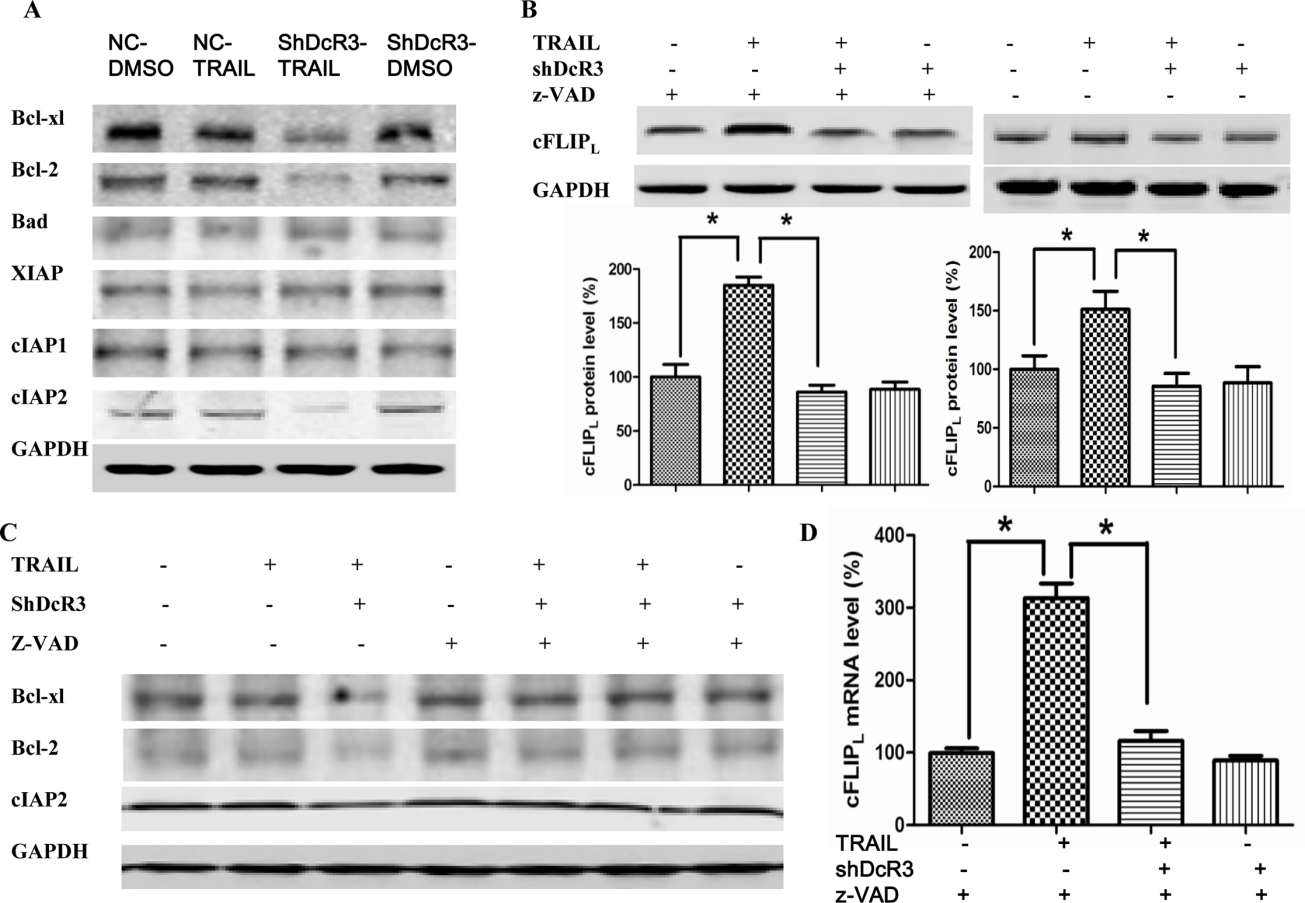
Effects of DcR3 knockdown on apoptosis-regulatory proteins. In the presence or absence of shDcR3 and z-VAD, Huh7 cells were treated with TRAIL (75ng/ml) for 24 h. (A-B) Western blots were used to determine the target proteins. (C) Western blot was used to detect the protein level of cFLIP_L_. (D) Real time PCR was used to detect the mRNA level of cFLIP_L_. Each value represents the mean ± SEM of three independent experiments performed in triplicate.*P<0.05 and **P<0.01.

It is known that cell apoptosis is regulated by pro- and anti-apoptotic proteins such as Bcl-2- and IAP-family proteins [21, 22]. Thus, we investigated the expression of these proteins after silencing DcR3 expression. We found that Bcl-xl and Bcl-2 protein expression was reduced following the addition of either shDcR3 or TRAIL to experimental cells as compared to mock cells; Bcl-xl and Bcl-2 protein expression even lower in cells treated with shDcR3 plus TRAIL (Fig 3A). For IAP-family proteins, we found that shDcR3/TRAIL decreases the cIAP-2 protein level but did not alter the cIAP-1 and XIAP protein level (Fig 3A). However, when cells were pretreated with z-VAD-fmk, a pan-caspase inhibitor, none of the decreased proteins were altered by treatment with shDcR3/TRAIL (Fig 3B). These results suggest that the protein alterations induced by shDcR3/TRAIL are due to caspase-dependent cleavage, not to a change in protein expression. Because cFLIP_L_ is a crucial competitive inhibitor of caspase-8 activation; its expression can regulate the sensitivity of tumor cells to TRAIL-mediated apoptosis. Therefore, we next examined the expression of cFLIP_L_ after different treatments. Our results suggested that treatment with TRAIL alone increased the expression of cFLIP_L_ in the presence or absence of z-VAD-fmk, but this effect was significantly reduced by co-treatment with shDcR3 (p<0.05; Fig 3C). However, in the absence of z-VAD-fmk, the expression of cFLIP_L_ was increased less than that in the presence of z-VAD-fmk indicating the proteolytic cleavages of cFLIP_L_ by caspase. We then examined the regulation of mRNA expression in cFLIP_L_ by shDcR3 using qPCR. Co-treatment with shDcR3 in Huh7 cells abrogated TRAIL-induced cFLIP_L_ mRNA increase (Fig 3D). Our results therefore suggest that the downregulation of DcR3 expression inhibits a TRAIL-induced increase in cFLIP_L_ expression at the transcriptional level.

### Downregulation of DcR3 enhanced TRAIL-induced apoptosis by affecting the NF-κB pathway

**Fig 4:**
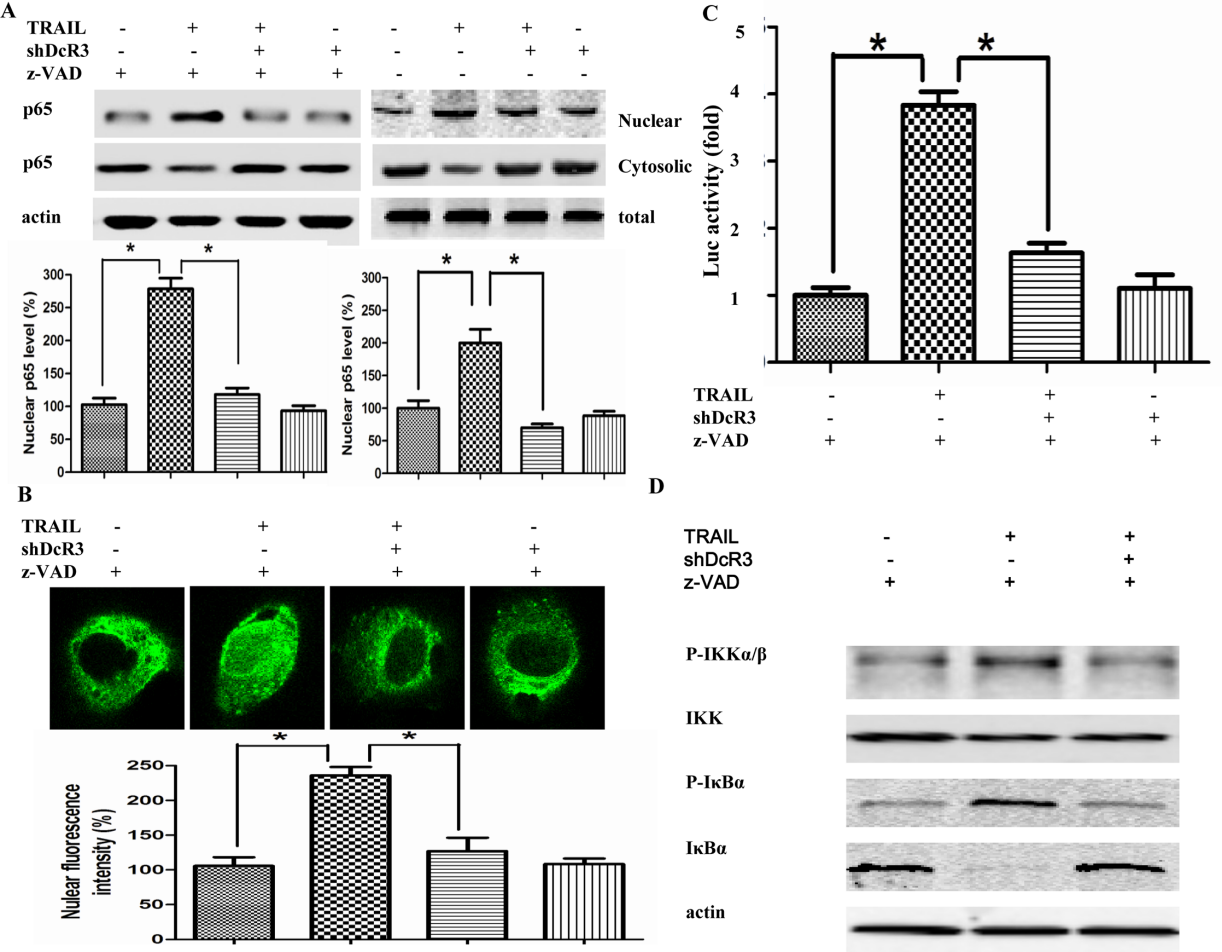
Knockdown of DcR3 inhibits TRAIL-induced NF-κB activation. After infection with shDcR3 for 12 h in the presence of 100 μ M z-VAD-fmk, Huh7 cells were treated with TRAIL (75ng/ml) for 24 h. (A) The levels of cytosolic and nuclear p65 proteins were determined by western blots. (B) The nuclear translocation of NF-κB p65 were examined by confocal microscopy. Original magnification ×400. (C) Luciferase activity was determined by a luminometer in cell lysates. (D) Western blot was used to determined the IKK α / β phosphorylation and I κ B phosphorylation/degradation. Each value represents the mean ± SEM of three independent experiments performed in triplicate.*P<0.05 and **P<0.01.

TRAIL has been shown to activate not only the apoptotic signal pathway but also the survival pathway through NF-κB -mediated cFLIP_L_ expression [12]. Moreover, studies have found that blocking the activation of NF-κB enhanced the sensitization of TRAIL-mediated apoptosis in cancer cells [23]. Accordingly, we analyzed the mechanisms associated with the enhancement of TRAIL-mediated apoptosis through DcR3 silencing. Then we examined the regulation of silencing DcR3 expression on TRAIL-induced NF-κB activation. First, the nuclear translation of the p65 subunit of NF-κB was detected by western blot. As shown in Fig 4A, the nuclear translation of the p65 subunit of NF-κB was increased in Huh7 cells after treatment with TRAIL in the presence or absence of z-VAD-fmk, but treatment with shDcR3 effectively suppressed these cellular events. However, in the absence of z-VAD-fmk, nuclear translation of the p65 was increased less than that in the presence of z-VAD-fmk indicating the proteolytic cleavages of NF-κB p65 by caspase. To avoid proteolytic cleavages of p65 by caspase, further studies we pretreated with Huh7 cells with z-VAD-fmk. The result of confocal microscopy was consistent with western blot (Fig 4B). We further examined the effects of shDcR3 on TRAIL-induced NF-κB transcriptional activity, as determined by NF-κB-responsive luciferase reporter assay. Consistent with the western and confocal results, the NF-κB reporter activity was significantly increased after TRAIL stimulation (p<0.05), while this increase was abolished by co-treatment with shDcR3 (Fig 4C).

As IKK-dependent phosphorylation and proteolytic degradation of IκBα has been reported to be located upstream of the NF-κB pathway [24], we investigated of the effect of shDcR3 on the phosphorylation of IKK and the degradation of IκBα in TRAIL-treated Huh7 cells. Increased IKK phosphorylation and IκBα degradation were observed in Huh7 cells after TRAIL stimulation (p<0.05), and treatment with shDcR3 suppressed this increases (Fig 4D).

### Inhibition of cFLIP_L_ expression sensitizes Huh7 cells to anti-proliferative and pro-apoptotic effects

**Fig 5:**
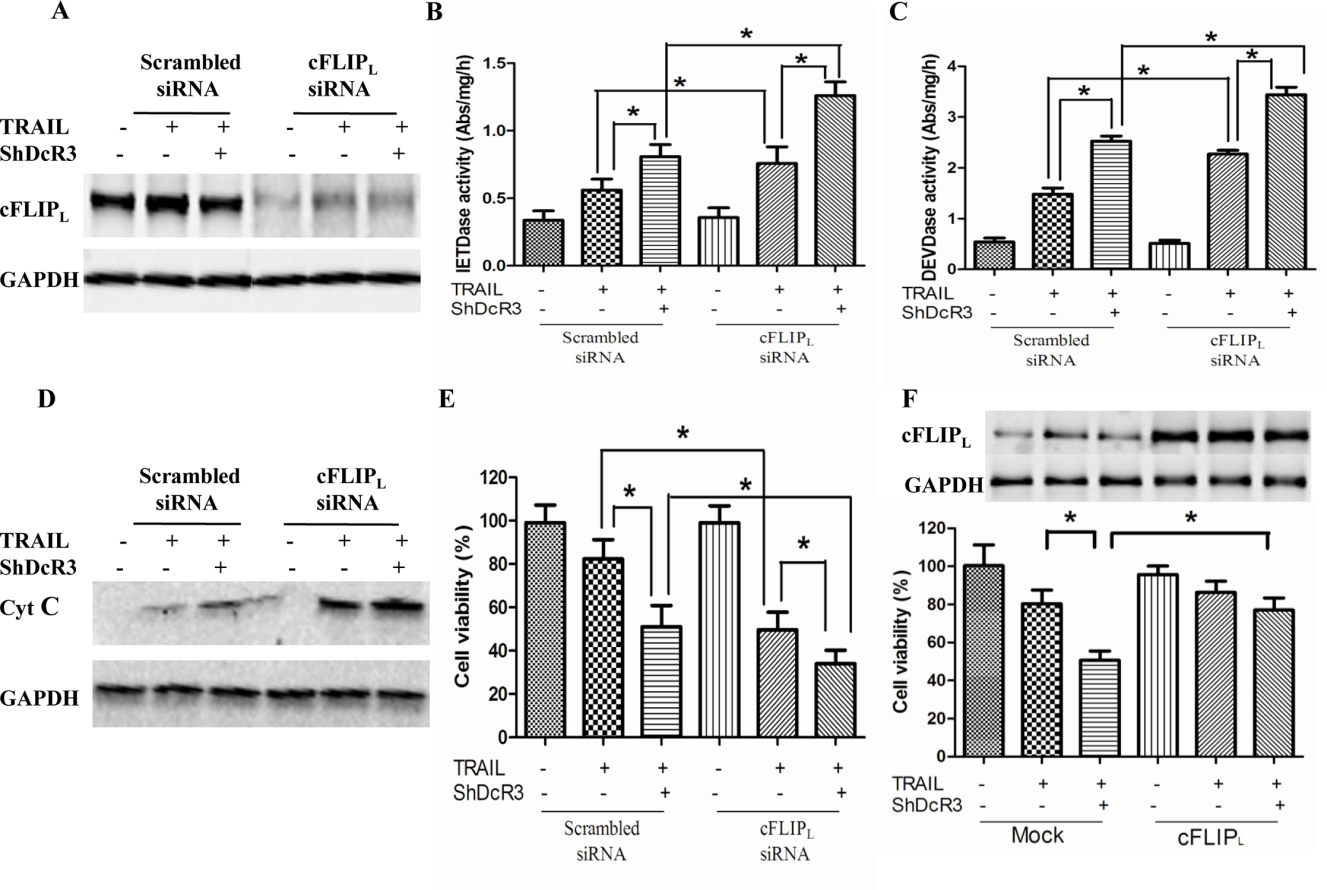
Inhibition of cFLIP_L_ expression sensitizes Huh 7 cells to anti-profiferative and pro-apoptotic effects. (A) Western blot was used to determine the cFLIP_L_ protein level after transfection with cFLIP_L_ siRNA for 24 h. (B-C) Transfected cells were treated with TRAIL in the presence or absence of shDcR3 for 24 h. IETDase colorimetric assay was used to determine the IETDase and DEVDase activities in cell lysates. (D) Western blot was used to detect the mitochondrial cytochrome c release. (E) CCK-8 assay was used to determine the cell viability. (F) Cells were transfected with mock or cFLIP_L_ vector for 24 h, cFLIP_L_ protein level was detected by western blot. In the presence or absence of shDcR3, transfected cells were treated with TRAIL for 4 h. CCK-8 was used to measure the cell viability. Each value represents the mean ± SEM of three independent experiments performed in triplicate. *P<0.05 and **P<0.01.

We used siRNA to knockdown cFLIP_L_ expression, and investigated its effect on shDcR3-mediated apoptosis in Huh7 cells treated with TRAIL. The expression of cFLIP_L_ was significantly decreased in Huh7 cells transfected with cFLIP_L_ siRNA as opposed to Huh7 cells transfected with scrambled siRNA (p<0.05; Fig 5A). Caspase-8/-3 activity and cytochorme c release in Huh7 cells treated with cFLIP_L_ siRNA plus TRAIL or shDcR3 or both were significantly higher than cells treated with scrambled siRNA (Fig 5B–5D). Our cell viability assay supported these results: Cells treated with cFLIP_L_ siRNA plus TRAIL or shDcR3 or both showed a significant increase in cell apoptosis compared with cells treated with scrambled siRNA (p<0.05; Fig 5E). Then we examined the effect of overexpression of cFLIP_L_ on the cell apoptosis in Huh 7 cells treated with TRAIL plus shDcR3. As shown in Fig 5F, cell apoptosis was significantly increased in Huh 7 cells when co-treated with TRAIL and shDcR3; however, overexpression of cFLIP_L_ effectively suppressed these increases. In sum, our data suggest that cFLIP_L_ expression regulates the cells apoptosis induced by TRAIL in Huh 7 cells, and downregulation of cFLIP_L_ by shDcR3 may be play an important role in shDcR3-mediated sensitization of TRAIL-induced cell apoptosis.

### ShDcR3 down-regulates cFLIP_L_ expression and inhibits NF-κB activation in HepG2 cells

**Fig 6:**
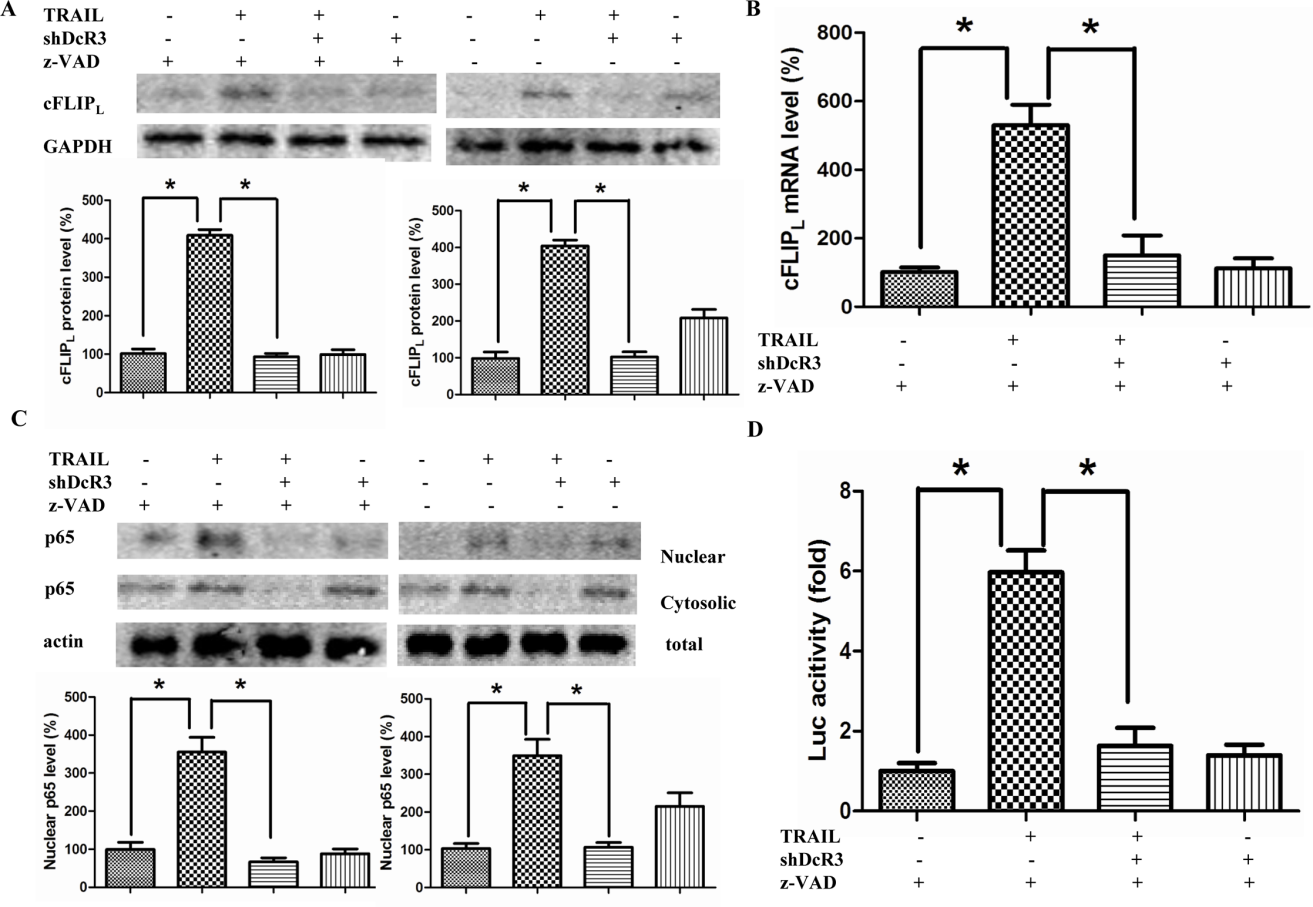
Knockdown of DcR3 inhibits TRAIL-induced cFLIP_L_ upreguation and NF-κB activation in HepG2 cells. **(A)** In the presence or absence of shDcR3 and z-VAD, HepG2 cells were treated with TRAIL (75ng/ml) for 24 h. Western blot was used to detect the protein level of cFLIP_L_. (B) Real time PCR was used to detect the mRNA level of cFLIP_L_. (C) After infection with shDcR3 for 12 h in the presence or absence of 100 μ M z-VAD-fmk, HepG2 cells were treated with TRAIL (75ng/ml) for 24 h. The levels of cytosolic and nuclear p65 proteins were determined by western blots. (D) Luciferase activity was determined by a luminometer in cell lysates. Each value represents the mean ± SEM of three independent experiments performed in triplicate.*P<0.05 and **P<0.01.

To explore whether the effect is specific for Huh7 cells, we first detected the effect of shDcR3 on the expression of cFLIP_L_ in the presence or absence of z-VAD-fmk in HepG2 cells. The results showed that treatment with TRAIL alone increased the expression of cFLIP_L_ in the presence or absence of z-VAD-fmk, but this effect was significantly reduced by co-treatment with shDcR3 in HepG2 cells (p<0.05; Fig 6A). We then examined the regulation of mRNA level in cFLIP_L_ by shDcR3 using qPCR. Co-treatment with shDcR3 in HepG2 cells also abrogated TRAIL-induced increase of cFLIP_L_ mRNA (Fig 6B). Next, we examined the regulation of silencing DcR3 expression on TRAIL-induced NF-κB activation. First, the nuclear translation of the p65 subunit of NF-κB was detected by western blot. As shown in Fig 6C, the nuclear translation of the p65 subunit of NF-κB was increased in HepG2 cells after treatment with TRAIL in the presence or absence of z-VAD-fmk, while treatment with shDcR3 effectively suppressed these cellular events. We further examined the effects of shDcR3 on TRAIL-induced NF-κB transcriptional activity in HepG2 cells, as determined by NF-κB-responsive luciferase reporter assay. Consistent with the results of western blot, the NF-κB reporter activity was significantly increased after TRAIL stimulation (p<0.05), while this increase was abolished by co-treatment with shDcR3 (Fig 6D). Therefore our results suggest that the downregulation of DcR3 expression also inhibits TRAIL-induced cFLIP_L_ expression and NF-κB activation in HepG2 cells.

## Discussion

DcR3 has been reported to regulate the tumor differentiation and apoptosis in a wide variety of tumors. It has been demonstrated that downregulation of DcR3 unmasked TRAIL and increased TRAIL-induced apoptosis in pancreatic cancer [25]. In HCC research, Wu et at found that reduction of DcR3 could increase TRAIL-induced apoptosis through upregulation of DR5 expression [26]. However, Zhang et al have reported that the expression of DR4 and DR5 was higher in the cancerous tissues than in the normal tissues in hepatocellular carcinoma. This indicated that other mechanisms might participate in TRAIL-specific sensitization. A small portion of researchers have revealed that the TRAIL-DR4/5 signal does not exclusively activate the apoptotic pathway leading to apoptosis, but also confers survival benefit upon cells in specific conditions probably with the implication of NF- κB. In this study we showed that silencing DcR3 expression with shRNA increased TRAIL-induced apoptosis and inhibited the proliferation of Huh7 cells. In addition, silencing DcR3 enhanced TRAIL-mediated caspase cascade. We further found that silencing DcR3 expression inhibited TRAIL-induced NF-κB activation and cFLIP_L_ expression. Furthermore, downregulation of cFLIP_L_ mimicked shDcR3-enhanced TRAIL-mediated apoptosis in Huh7 cells, and overexpression of cFLIP_L_ inhibited shDcR3-enhanced TRAIL-mediated apoptosis. These results suggested that shDcR3 enhanced TRAIL-induced cell apoptosis through activation of the extrinsic apoptotic cascade and suppression of TRAIL-mediated NF-κB activation and subsequent anti-apoptotic cFLIP_L_ expression. Our results indicate that inhibition of DcR3 is a potent therapeutic goal, as this may overcome the resistance of Huh7 cells to TRAIL-induced apoptosis. To explore whether the effect is specific to Huh 7 cells, we also examined the effect of shDcR3 on the expression of cFLIP_L_ and the activation of NF-κB in HepG2 cells. The results are consistent with those in Huh7 cells.

TRAIL, a novel member of the TNF superfamily, differs from TNF and Fas. It only acts on virus-infected cells, transformed cells, and tumor cells. Due to its selective cytotoxicity to tumor cells, TRAIL is an attractive target for antitumor therapy [27]. Although TRAIL can strongly induce apoptosis in various types of tumor cells, most HCC cells are resistant to TRAIL-induced apoptosis [28]. Due to the extremely limited success of TRAIL monotherapy, a combination therapy with TRAIL has been used to treat hepatoma [29]. Shin et al. reported that TRAIL had little effect on HCC cells, while a combination of cisplatin and TRAIL significantly altered the sensitivity of HCC cells to TRAIL-induced apoptosis via the activation of the mitochondrial pathway and the amplification of the TRAIL-mediated death receptor pathway [30]. Other chemotherapeutic agents, including antimycin, cisplatin, paclitaxel, and 5-FU were also reported to act synergistically with TRAIL [31–33]. The underlying mechanisms include the activation of caspase-8, the increased aggregation of DISC, the upregulation of DR5, and the inhibition of NF-κB [31, 34–36]. The expression of pro- and anti-apoptotic proteins is known to regulate cell apoptosis, [21]. Bcl-xl and Bcl-2 are anti-apoptotic members of the Bcl-2 protein family, and this family has been shown to play a vital role in the regulation of the apoptosis of HCC cells [37]. Overexpression of Bcl-xl and Bcl-2 contributes to TRAIL resistance in various cancers, including HCC [38]. Bad belongs to the pro-apoptotic Bcl-2 family, and it can increase mitochondrial cytochrome c release and induce cell apoptosis [39]. Upregulation of Bad sensitizes colon cancer cells to TRAIL-induced apoptosis [40]. Therefore the regulation of Bcl-2 family proteins can sensitize cells to TRAIL-mediated apoptosis. Here, we found that Bcl-xl and Bcl-2 protein expression was reduced and that Bad protein expression was increased in Huh7 cells co-treated with TRAIL and shDcR3. However, no alterations were observed when the cells were pretreated with pan-caspase inhibitors, indicating that protein alterations were due to proteolytic cleavage by caspase [41]. These results suggest that Bcl-2 family proteins are not involved in shDcR3-enhanced TRAIL-mediated apoptosis in Huh7 cells.

Similarly, the proteins in the inhibitor of apoptosis (IAP) protein family including cIAP1, cIAP2, and XIAP, have been reported to be important factors in determining the apoptosis of HCC cells [22]. It has been shown IAP proteins that involved in the sensitivity of tumor cells to TRAIL-induced apoptosis [42, 43]. We also measured the expression of these proteins after different treatments. The alteration of IAP protein expression were similar to that of Bcl-2 protein: downregulation of DcR3 did not regulate the expression of IAP proteins. This indicates that IAP family proteins are not involved in shDcR3-enhanced TRAIL-mediated apoptosis in Huh7 cells.

TRAIL has been shown to induce two different signals: caspase-mediated cell death and NF-κB-mediated gene induction [44]. Through inhibition of NF-κB activation, TRAIL-induced cell apoptosis was significantly increased [42]. cFLIP_L_, an anti-apoptotic protein, can be upregulated by NF-κB activation [10]. Kang et al. demonstrated that the silencing cFLIP_L_ expression increased TRAIL-induced apoptosis in tumor cells [45]. Previous studies have demonstrated that DcR3 can increase monocyte adhesion through NF-κB-mediated upregulation of the intercellular adhesion molecule vascular cell adhesion protein [46]. Here, we found that downregulation of DcR3 expression abolished TRAIL-induced NF-κB activation and cFLIP_L_ expression. These results indicated that downregulation of DcR3 expression by shRNA inhibited TRAIL-induced NF-κB activation and cFLIP_L_ expression, as well as increased TRAIL-induced caspase cascade and apoptosis. Furthermore, downregulation of cFLIP_L_ mimicked shDcR3-enhanced TRAIL-mediated apoptosis in Huh7 cells, and overexpression of cFLIP_L_ inhibited shDcR3-mediated TRAIL sensitization. However, downregulation of cFLIP_L_ did not have as great an effect as we had expected, which implies that other factors were involved in inhibiting TRAIL-induced apoptosis in these cells as was also suggested by Onco Targets Ther. 2017 Jan 18;10:417–428. Our results indicate that shDcR3 inhibits the growth of HCC cells by suppressing NF-κB dependent cFLIP_L_ expression, as well as inducing caspase-dependent apoptosis.

IKK-dependent phosphorylation and proteolytic degradation of IκBαwere reported located upstream of the NF-κB pathway. Here, we found that downregulation of DcR3 inhibited both IKK phosphorylation and IκBα degradation, as well as NF-κB activation in Huh7 cells pretreated with z-VAD. Although the underlying mechanism of the inhibition of IKK by shDcR3 is not known, downregulation of DcR3 is likely to inhibit the extracellular signal-regulated kinase ribosomal s6 kinase signal pathway, which results in the suppression of NF-κB activation and subsequent cFLIP_L_ expression. cFLIP_L_ is reported to be an important anti-apoptotic protein. cFLIP_L_ prevents the recruitment of procaspase-8 to DISC through forming heterodimeric complex with procaspase-8, resulting in the suppression of caspase-8 activtion and downstream apoptosis cascade. Downregulation of cFLIP_L_ by shDcR3 rendered Huh7 cells sensitive to TRAIL-induced apoptosis and was accompanied with the caspase-8 activation and downstream signal propagation. Thus, our data suggest that NF-κB-mediated cFLIP_L_ expression seems to play a role in shDcR3-mediated TRAIL-sensitization.

In conclusion, DcR3 is a vital mediator of HCC cell sensitivity to TRAIL-induced apoptosis. Downregulation of DcR3 through shRNA reversed HCC resistance to TRAIL by inhibiting the NF-κB pathway, specifically cFLIP_L_ expression. Therefore, shDcR3 affects the sensitization of HCC cells to TRAIL-induced apoptosis, and thus could be an attractive strategy for promoting HCC cell death.

## Supporting information

S1 Fig Expression of DcR3 in HCC cells. (A) DcR3 protein expression in the four cell lines was detected by western blots. (B) DcR3 mRNA levels in the four cell lines were detected by real-time PCR. (C) The levels of DcR3 secreted into the supernatants in the four cell lines were detected by ELISA. (D) After infection with shDcR3 for 24 h, DcR3 protein was detected by western blots in Huh7 cells. (E) After infection with shDcR3 for 24 h, DcR3 protein was detected by western blots in HepG2 cells. Each value represents the mean ± SEM of three independent experiments performed in triplicates.*P<0.05, compared with Chang liver cells.
(TIF)

S2 Fig Knockdown of DcR3 on TRAIL-induced apoptosis in the HepG2 and Huh-7 cell lines. Cells were divided into four groups and incubated in NC-DMSO (control), shDcR3-DMSO, NC-TRAIL, and shDcR3-TRAIL for 24 h. (A) Flow cytometry was used to analyze cell apoptosis in the four groups in both Huh7 cells and HepG2 cells. (B–C) The marker protein of apoptosis, PARP, was detected by western blots in the four groups both in Huh7 and HepG2 cells. *P<0.05, compared with mock cells in Huh7 cells; ^#^ P<0.05, compared with mock cells in HepG2 cells.
(TIF)
