# Supplementary figures and images for: ShDcR3 sensitizes TRAIL-resistant HCC cells by inducing caspase-dependent apoptosis while suppressing NF-κB dependent cFLIPL expression
Source: PLoS One. 2018 Feb 14;13(2):e0191545. doi: 10.1371/journal.pone.0191545 (PMC5812574; doi:10.1371/journal.pone.0191545)

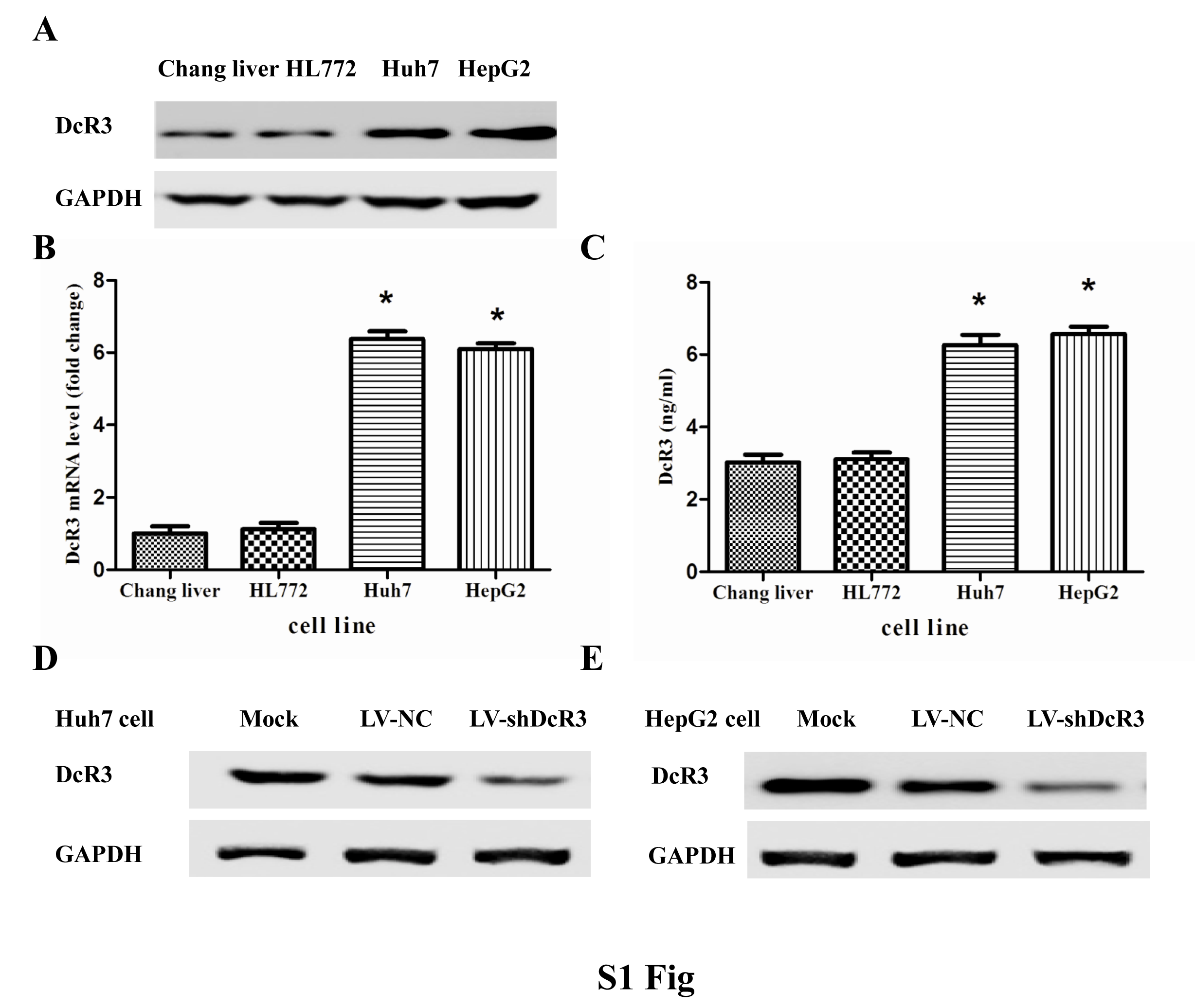

Supplement: S1 Fig — (A) DcR3 protein expression in the four cell lines was detected by western blots. (B) DcR3 mRNA levels in the four cell lines were detected by real-time PCR. (C) The levels of DcR3 secreted into the supernatants in the four cell lines were detected by ELISA. (D) After infection with shDcR3 for 24 h, DcR3 protein was detected by western blots in Huh7 cells. (E) After infection with shDcR3 for 24 h, DcR3 protein was detected by western blots in HepG2 cells. Each value represents the mean ± SEM of three independent experiments performed in triplicates.*P<0.05, compared with Chang liver cells. (TIF) [file pone.0191545.s001.tif]

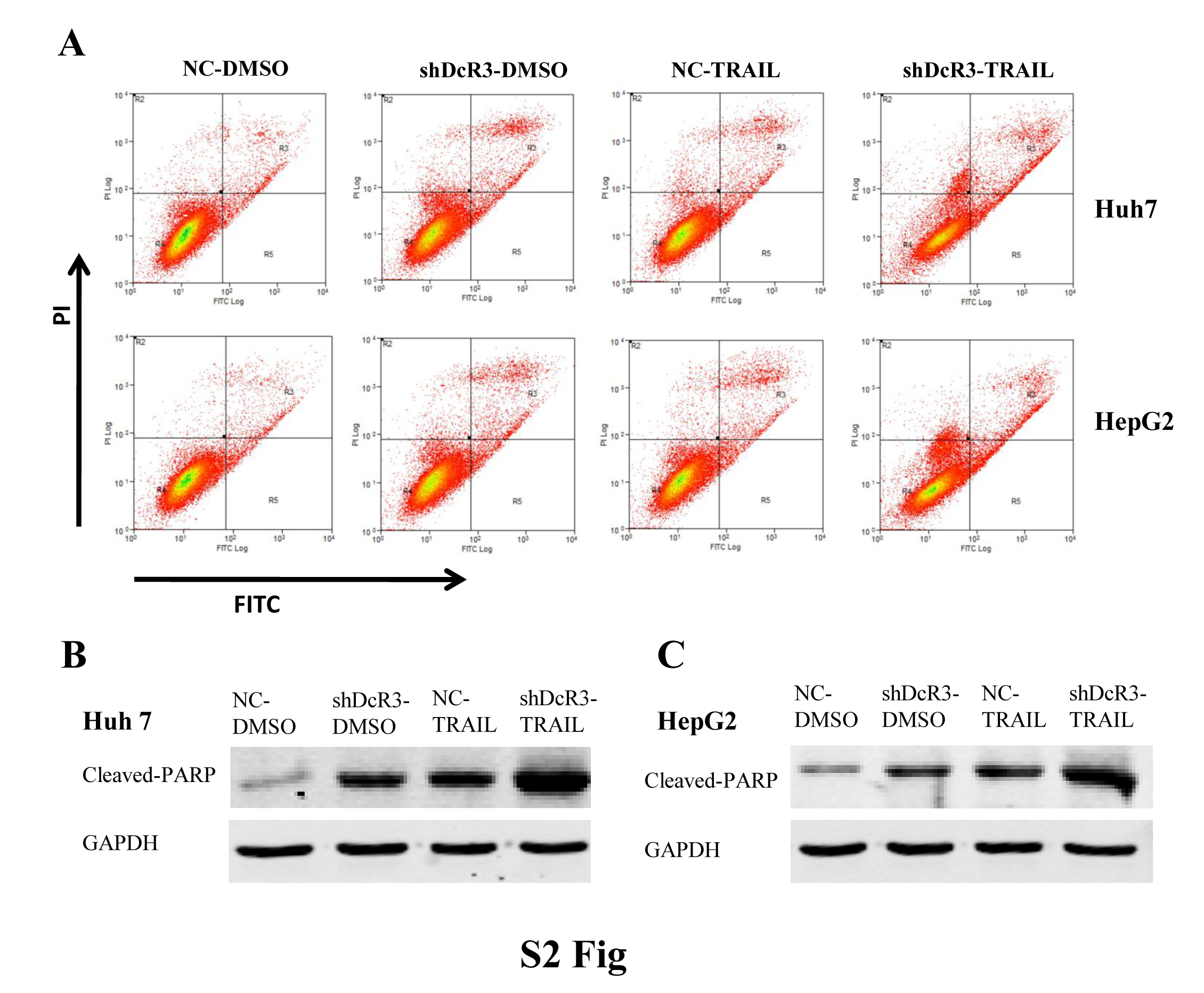

Supplement: S2 Fig — Cells were divided into four groups and incubated in NC-DMSO (control), shDcR3-DMSO, NC-TRAIL, and shDcR3-TRAIL for 24 h. (A) Flow cytometry was used to analyze cell apoptosis in the four groups in both Huh7 cells and HepG2 cells. (B–C) The marker protein of apoptosis, PARP, was detected by western blots in the four groups both in Huh7 and HepG2 cells. *P<0.05, compared with mock cells in Huh7 cells; # P<0.05, compared with mock cells in HepG2 cells. (TIF) [file pone.0191545.s002.tif]
